# Predicting the Recurrence of Hepatocellular Carcinoma after Primary Living Donor Liver Transplantation Using Metabolic Parameters Obtained from ^18^F-FDG PET/CT

**DOI:** 10.3390/jcm11020354

**Published:** 2022-01-12

**Authors:** Sungmin Kang, Joo Dong Kim, Dong Lak Choi, Byungwook Choi

**Affiliations:** 1 Department of Nuclear Medicine, Daegu Catholic University Medical Center, Daegu Catholic University School of Medicine, 33, Duryugongwon-ro 17-gil, Nam-gu, Daegu 42472, Korea; kufa77@cu.ac.kr; 2 Division of Hepatobiliary Pancreas Surgery and Abdominal Organ Transplantation, Department of Surgery, Daegu Catholic University Medical Center, Daegu Catholic University School of Medicine, 33, Duryugongwon-ro 17-gil, Nam-gu, Daegu 42472, Korea; milledr@cu.ac.kr (J.D.K.); dnchoi@cu.ac.kr (D.L.C.)

**Keywords:** ^18^F-FDG PET/CT, hepatocellular carcinoma, living donor liver transplantation, prognosis, recurrence, standardized uptake value normalized by lean body mass

## Abstract

This study evaluated the prognostic value of metabolic parameters based on the standardized uptake value (SUV) normalized by total body weight (bwSUV) and by lean body mass (SUL) measured on ^18^F-fluorodeoxyglucose positron emission tomography/computed tomography (18F-FDG PET/CT) for predicting tumor recurrence after primary living donor liver transplantation (LDLT) in patients with hepatocellular carcinoma (HCC) without transplantation locoregional therapy. This retrospective study enrolled 49 patients with HCC. The maximum tumor bwSUV (T-bwSUVmax) and SUL (T-SULmax) were measured on PET. The maximum bwSUV (L-bwSUVmax), mean bwSUV (L-bwSUVmean), maximum SUL (L-SULmax), and mean SUL (L-SULmean) were measured in the liver. All metabolic parameters were evaluated using survival analyses and compared to clinicopathological factors. Tumor recurrence occurred in 16/49 patients. Kaplan–Meier analysis revealed that all metabolic parameters were significant (*p* < 0.05). Univariate analysis revealed that prothrombin-induced by vitamin K absence or antagonist-II; T-stage; tumor number; tumor size; microvascular invasion; the Milan criteria, University of California, San Francisco (UCSF), and up-to-seven criteria; T-bwSUVmax/L-bwSUVmean; T-SULmax; T-SULmax/L-SULmax; and T-SULmax/L-SULmean were significant predictors. Multivariate analysis revealed that the T-SULmax/L-SULmean (hazard ratio = 115.6; *p* = 0.001; cut-off, 1.81) and UCSF criteria (hazard ratio = 172.1; *p* = 0.010) were independent predictors of tumor recurrence. SUL-based metabolic parameters, especially T-SULmax/L-SULmean, were significant, independent predictors of HCC recurrence post-LDLT.

## 1. Introduction

The incidence of hepatocellular carcinoma (HCC), which accounts for the majority of primary liver cancer and occurs mostly in the setting of chronic liver disease and cirrhosis, has risen in recent years [1]. Orthotopic liver transplantation (LT) has been established as the standard treatment for select candidates with underlying liver cirrhosis (LC) because it removes the tumor with the widest possible surgical margins and tumor-generating cirrhotic background liver simultaneously [2]. Although living donor LT (LDLT) has been established as an alternative and effective treatment for HCC, especially in Asia, countries in the Americas and Europe are still distressed by long waiting times for deceased donor liver transplantation (DDLT) [2,3,4,5]. The risk of tumor recurrence that occurs in approximately 6–20% of patients is another concern accompanied by the use of LT for HCC, which represents not only the loss of the donor organ but is also associated with a poor prognosis [6,7,8,9]. The current selection criteria including the Milan criteria; University of California, San Francisco (UCSF); and up-to-seven criteria that utilize the number and size of the tumor have been proposed for the selection of HCC patients for LT, and have demonstrated excellent long-term outcomes comparable to those for LT in non-malignant disease [10,11,12]. However, these histopathological prognostic factors can reliably assess only the explanted liver and not the clinical features available before LT. Moreover, the evaluation of the number and size of the tumor with pre-LT radiographic imaging studies is limited by the high risk of the under- and overestimation of the tumor burden [13,14,15].

Standardized uptake value (SUV), which can be obtained from ^18^F-fluorodeoxyglucose positron emission tomography/computed tomography (^18^F-FDG PET/CT), is a widely accepted semiquantitative metabolic parameter and normalized using the total body weight (bwSUV), lean body mass (SUL), and body surface area [16]. Recently, bwSUV-based metabolic parameters have been proven to be useful in the prediction of tumor recurrence after LT for HCC [17,18,19,20,21,22]. However, it has been reported that the bwSUV could overestimate metabolic activity in overweight/obese patients, because heavy patients have a relatively higher proportion of fat in their bodies than light patients, and fat has low ^18^F-FDG uptake in the fasting state [23]. Furthermore, a recent study reported that the maximum bwSUV (bwSUVmax) and mean bwSUV (bwSUVmean) in the liver were significantly higher in patients with obesity than those with a normal body mass index (BMI) [24]. Thus, the authors alternatively recommended using the SUL-based parameters because the maximum SUL (SULmax) and mean SUL (SULmean) since these factors did not differ significantly from each other in their study. Therefore, it is expected that SUL-based semiquantitative metabolic parameters could also serve as effective predictors of tumor recurrence post-LT. However, most previous studies used bwSUV-based semiquantitative metabolic parameters, and data based on the evaluation of the prognostic value of SUL in patients before LT are scarce [25].

Studies have demonstrated the survival benefits of pre-LT bridging locoregional therapy (LRT), such as transcatheter arterial chemoembolization (TACE), radiofrequency ablation (RFA), and percutaneous ethanol injection therapy (PEIT) [26,27]. Furthermore, studies have reported a change in the tumoral uptake of ^18^F-FDG after pre-LT LRT [28,29,30]. Therefore, a separate evaluation of the prognostic value of metabolic parameters would seem to be more appropriate in patients who underwent primary LT and those who received LT after LRT. However, in previous studies, whether LRT was performed before and after ^18^F-FDG PET/CT, and the time interval between ^18^F-FDG PET/CT and LRT, was unclear [19,20,21,22]. Furthermore, data on the prognostic value of metabolic parameters that can predict tumor recurrence after primary LT remain insufficient. In contrast, this study focused on patients who received primary LDLT due to its increasing application.

The aims of this retrospective study were to investigate the prognostic value of bwSUV- and SUL-based metabolic parameters obtained using ^18^F-FDG PET/CT for the prediction of tumor recurrence after primary LDLT and compare them with other clinicopathological factors.

## 2. Materials and Methods

### 2.1. Patients

**Figure 1:**
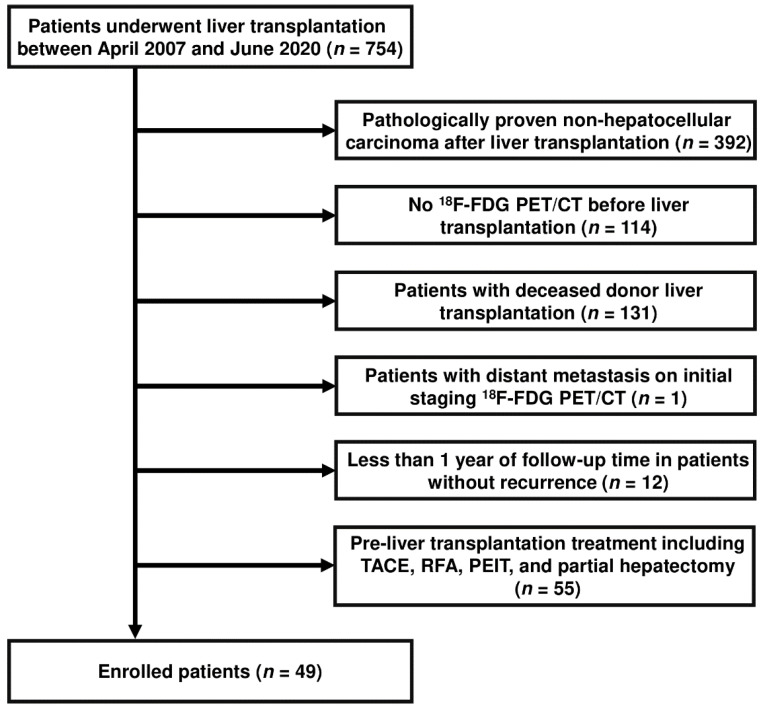
Flow chart for inclusion and exclusion of patients in this retrospective study.

Patients who underwent LDLT for HCC at our institution between April 2007 to June 2020 were evaluated retrospectively in this study. The inclusion criteria were as follows: (1) pathological confirmation of HCC after LDLT, (2) ^18^F-FDG PET/CT scan performed within 3 months before LDLT, (3) no pre-LT treatment including TACE, PEIT, RFA, and hepatectomy before and after the ^18^F-FDG PET/CT scan, (4) post-LT follow-up duration of more than 12 months in case of absence of recurrence. We excluded all DDLT patients in in our hospital because none of these patients met the inclusion criteria. The flowchart of the patient selection process is shown in Figure 1. 

At our institution, the essential policy for the selection of recipients for LDLT from among patients with HCC is based on the application of the UCSF criteria to preoperative imaging studies such as contrast-enhanced CT (CECT), magnetic resonance imaging (MRI), or ^18^F-FDG PET/CT. However, LDLT was also performed for patients who did not meet the UCSF criteria when a patient and his/her family strongly desired LT, and there was no evidence of major vascular invasion or extrahepatic metastasis on imaging studies. For post-LT management, patients stayed in the hospital for around three to six weeks after LDLT and asked for regular outpatient visits after discharge. To prevent complications of organ transplantation, all patients had taken immunosuppressant drugs (tacrolimus and mycophenolate mofetil). In addition, patients who had hepatitis B before LDLT were prescribed regular injections of hepatitis B antibodies to prevent hepatitis B relapse after surgery. Routine post-LT surveillance was based on CECT, which was conducted every 3 months during the first year after LT and every 6 months thereafter. Ultrasonography and MRI were employed complementarily in some cases. Blood investigations of the hepatic and tumor markers were conducted every 2–3 months. Tumor recurrence was confirmed by imaging studies. Recurrence-free survival (RFS) was defined as the time interval between LDLT and confirmation of recurrence. We reviewed the medical records for clinicopathological factors including age, sex, model for end-stage liver disease (MELD) score, Child–Pugh score, BMI, etiology, status of cirrhosis, presence of ascites, pre-LT serum alpha-fetoprotein (AFP), pre-LT serum prothrombin-induced by vitamin K absence or antagonist-II (PIVKA-II), T-stage, tumor number, largest tumor size (cm), microvascular invasion (MVI), Milan criteria, UCSF criteria, and up-to-seven criteria.

Our institutional review board approved this study, and the need for obtaining written informed consent from the participants was waived due to its retrospective design (IRB No. CR–21–055).

### 2.2. ^18^F-18 FDG PET/CT Acquisition

^18^F-FDG PET/CT scan was performed using the Discovery ST or Discovery IQ (GE Healthcare, Milwaukee, WI, USA) PET/CT scanner. All patients fasted for at least 6 h before the ^18^F-FDG injection, and each patient’s blood glucose concentration was confirmed to be <150 mg/dL. All patients with diabetes were asked to discontinue the anti-hyperglycemic drugs 12 h before the scan. Patients were administered 7.0 (Discovery ST) and 4.0 MBq/kg (Discovery IQ) of ^18^F-FDG intravenously, according to the PET/CT system. After 1 h of ^18^F-FDG uptake, an initial low-dose non-contrast CT scan was obtained for attenuation correction and localization. Immediately after the CT scan, standard PET images were acquired from the base of the skull or top of the brain to the proximal thigh. Both Discovery ST and Discovery IQ PET/CT scanners acquired images with a slice thickness of 3.75 mm for CT and 3.26 mm for PET. The transaxial field-of-view of the Discovery ST and Discovery IQ PET/CT scanners were 600 and 500 mm, and the matrix size was 128 × 128 and 256 × 256, respectively. The PET images were reconstructed using the ordered subset expectation–maximization iterative algorithm with 20 subsets and two iterations or Q Clear.

### 2.3. ^18^F-FDG PET/CT Interpretation and Image Analysis

**Figure 2:**
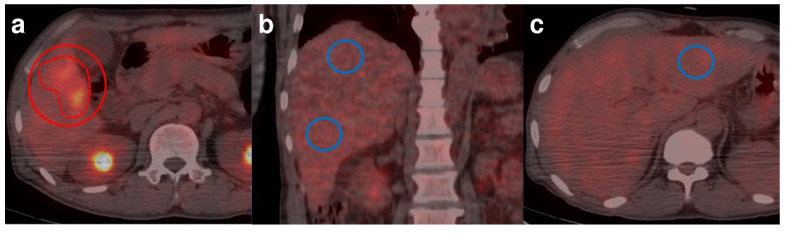
Measurement methods on ^18^F-fluorodeoxyglucose positron emission tomography/computed tomography images. (**a**) A spheric volume of interest (VOI) was carefully drawn to include the entire tumor (solid line), and the tumor contour (dashed line) was automatically drawn using a specific threshold of 41% of the maximum value. Then the maximum standardized uptake value (SUV) normalized by total body weight and the maximum SUV normalized by lean body mass was obtained. (**b**,**c**). For the background-liver regions, three spherical VOIs (14 cm^3^) of 30-mm diameter (blue circle) were drawn at 3 sites of the liver, 2 above and below the right portal vein, and 1 at the middle level of the left lobe, in a region where the tumor was not detected on other images.

All ^18^F-FDG PET/CT images were retrospectively reviewed on a dedicated vendor-supplied workstation (GE Advantage Workstation version 4.7 and Xeleris Workstation version 3.0, GE Healthcare). Two experienced nuclear medicine physicians interpreted the ^18^F-FDG PET/CT images of all patients until a consensus was reached. The HCCs were classified as positive (discernible hypermetabolic activity in the background liver) or negative (hypermetabolic activity not discernible in the background liver) for visual analysis. For semiquantitative analysis, we drew the volume of interest (VOI) for the tumor and background liver and measured the bwSUV and SUL in each VOI simultaneously. All tumor and background-liver regions were carefully defined with CECT or MRI scans of the liver. The VOI was drawn to encompass the highest activity of each tumor, by referring to the CT images of PET/CT, MRI, or other additional diagnostic CECT scans. The bwSUVmax of the tumor (T-bwSUVmax) and SULmax of the tumor (T-SULmax) were measured for each neoplastic lesion. The highest values of T-bwSUVmax and T-SULmax were recorded for each patient in case of multiple tumors. For the background-liver region, three spherical VOIs (14 cm^3^) of 30-mm diameter were drawn at 3 sites of the liver, 1 above and 1 below the right portal vein, and 1 at the middle level of the left lobe, in a region where the tumor was not detected on the other images (Figure 2). The bwSUVmax (L-bwSUVmax) and SULmax (L-SULmax) of the liver were defined as the highest bwSUVmax and SULmax of the 3 VOIs drawn on the background liver, and the bwSUVmean (L-bwSUVmean) and SULmean (L-SULmean) of the background liver were defined as the average value of the bwSUVmean and SULmean of the 3 VOIs.

### 2.4. Data Analyses

The numerical data were expressed as the mean ± standard deviation or median and range. We evaluated the following metabolic parameters for each patient to determine the most effective prognostic factor: the T-bwSUVmax, the ratio of T-bwSUVmax to L-bwSUVmax (T-bwSUVmax/L-bwSUVmax), the ratio of T-bwSUVmax to L-bwSUVmean (T-bwSUVmax/L-bwSUVmean), T-SULmax, the ratio of T-SULmax to L-SULmax, and the ratio of T-SULmax to L-SULmean (T-SULmax/L-SULmean). The area under the receiver operating characteristic curve (AUC) analysis was performed to determine the cut-off values for the prediction of recurrence. The prognostic value of the clinicopathological factors including age, sex, MELD score, Child–Pugh score, BMI, viral infection status, presence of LC, presence of ascites, pre-LT AFP level, PIVKA-II level, T-stage, tumor number, largest tumor size, MVI, Milan criteria, UCSF criteria, and up-to-seven criteria was analyzed.

The metabolic parameters and clinicopathological factors were compared using the chi-squared or Fisher’s exact test for categorical variables, and Student’s *t*-test, the Mann–Whitney U test, paired sample *t*-test, and Wilcoxon signed-rank test were used for the comparison of continuous variables. Kaplan–Meier analysis was conducted with the log-rank test using the metabolic parameters for the prediction of RFS. Univariate and multivariate analyses were also performed using the Cox proportional-hazards model with enter method for the prediction of recurrence. A parameter was included in the multivariate analysis if the *p*-value was <0.05 from the univariate analysis. The multicollinearity among significant clinicopathological factors and metabolic parameters on univariate analysis was evaluated using Pearson correlation coefficient before the multivariate analysis. All statistical analyses were performed using the IBM Statistical Package for the Social Sciences for Windows version 26.0 (IBM Corp., Armonk, NY, USA) and MedCalc Statistical Software version 20.009 (MedCalc Software Ltd., Ostend, Belgium; https://www.medcalc.org; accessed on 2021). *p*-values < 0.05 were considered statistically significant.

## 3. Results

### 3.1. Patient Characteristics

A total of 49 patients (40 men and 9 women; mean age 54 ± 6 years, range 41–65 years) were enrolled in this study. Their clinicopathological characteristics are summarized in Table 1. ^18^F-FDG PET/CT was performed 22.2 ± 15.4 days (range 2–70 days) before LDLT. The mean follow-up duration was 47.3 ± 36.0 months (range: 5–134 months). HCC recurred in 16 patients (32.7%) after 18.6 ± 14.6 months (5–54 months) of LDLT; 5 patients experienced recurrence within 12 months of LDLT, 7 patients between 12–24 months, and 4 patients between 24–60 months. Seven patients had extrahepatic recurrence, with the most common sites being lung (4 patients), bones (3 patients), peritoneum (1 patient), adrenal gland (1 patient), and regional lymph node (1 patient). Six patients had only hepatic recurrence, and 3 patients had both hepatic and extrahepatic (lung, 3 patients) recurrence.

### 3.2. Comparison between bwSUV and SUL According to the BMI and Liver Cirrhosis

The bwSUV and SUL values of the tumor and liver in each patient were compared using the paired *t*- and Wilcoxon signed-rank tests, and bwSUV was significantly higher than the corresponding SUL values in the entire study population, with respect to the BMI (Table 2).

The T-bwSUVmax was significantly higher than the T-SULmax in both BMI ≤ 25 and >25 groups. Meanwhile, the bwSUV values of the liver were also significantly higher than the corresponding SUL values, and the differences were greater in the BMI > 25 group. The difference between the respective bwSUV and SUL values was more prominent in the liver compared to the tumor.

Comparing the liver bwSUV and SUL parameters between the BMI ≤ 25 and >25 groups using the Mann–Whitney U test, there were no significant differences according to the BMI (all *p* > 0.05, respectively).

The comparison of the metabolic parameters between the LC and non-LC groups revealed no significant differences in the average L-bwSUVmax (2.61 ± 0.47 vs. 2.48 ± 0.47), L-bwSUVmean (2.09 ± 0.54 vs. 1.92 ± 0.47), L-SULmax (2.22 ± 0.41 vs. 2.05 ± 0.43), and L-SULmean (1.65 ± 0.41 vs. 1.45 ± 0.38) (all *p* > 0.05, respectively).

### 3.3. Metabolic Parameters on ^18^F-FDG PET/CT and Recurrence

Eight (44.4%) of the 18 PET-positive patients (according to the visual analysis) experienced recurrence, and the association between tumor recurrence and PET-positivity was not significant. According to the semiquantitative analysis, the bwSUV-based parameters, including T-bwSUVmax, T-bwSUVmax/L-bwSUVmax, and T-bwSUVmax/L-bwSUVmean, did not differ significantly between the non-recurrence and recurrence groups. Significant differences were observed between the SUL-based parameters, such as T-SULmax, T-SULmax/L-SULmax, and T-SULmax/L-SULmean, between the non-recurrence and recurrence groups. These results are summarized in Table 3.

T-bwSUVmax and T-bwSUVmax/L-bwSUVmax were not significant parameters for the prediction of recurrence, according to the receiver operating characteristic curve analysis. However, the T-bwSUVmax/L-bwSUVmax, T-SULmax, T-SULmax/L-SULmax, and T-SULmax/L-SULmean were significantly predictive of recurrence (*p* = 0.047, 0.003, <0.001, and <0.001, respectively). The T-SULmax/L-SULmean showed the highest AUC of 0.850 from among the metabolic parameters, with a sensitivity and specificity of 87.5% and 78.8%, respectively, at a cut-off value of 1.81. These results are summarized in Table 4.

### 3.4. Univariate and Multivariate Survival Analyses

**Figure 3:**
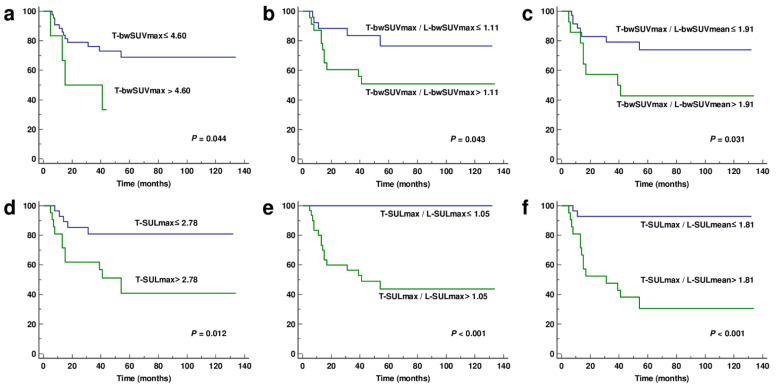
Kaplan–Meier analysis of recurrence-free survival according to metabolic parameters. Sixteen of the 49 patients experienced tumor recurrence during the follow-up period. (**a**) T-bwSUVmax, (**b**) T-bwSUVmax/L-bwSUVmax, (**c**) T-bwSUVmax/L-bwSUVmean, (**d**) T-SULmax, (**e**) T-SULmax/L-SULmax, and (**f**) T-SULmax/L-SULmean. T-, tumor; L-, liver; SUV, standardized uptake value; bwSUV, SUV normalized by total body weight; bwSUVmax, maximum value of bwSUV; bwSUVmean, mean value of bwSUV; SUL, SUV normalized by lean body mass; SULmax, maximum value of SUL; SULmean, mean value of SUL.

Kaplan–Meier analysis with the log-rank test revealed that the metabolic parameters were significant factors for the prediction of recurrence (*p* < 0.05 for all; Figure 3).

PIVKA-II (*p* = 0.026), T-stage (*p* = 0.001), tumor number (*p* = 0.035), largest tumor size (*p* = 0.013), MVI (*p* = 0.003), Milan criteria (*p* = 0.009), UCSF criteria (*p* = 0.001), up-to-seven criteria (*p* = 0.017), T-bwSUVmax/L-bwSUVmax (*p* = 0.040; cut-off 1.91), T-SULmax (*p* = 0.019; cut-off 2.78), and T-SULmax/L-SULmean (*p* = 0.001; cut-off 1.81) were significant metabolic and clinicopathological factors for the prediction of recurrence, as per the univariate Cox regression analysis. In correlation analysis, there were significant correlations between tumor factors including T-stage, tumor size, and tumor number and Milan, UCSF, and up-to-seven criteria (all *p* < 0.05). The AUCs of T-stage, tumor size, tumor number, Milan criteria, UCSF criteria, and up-to-seven criteria were 0.720 (95% CI, 0.573–0.839), 0.627 (95% CI, 0.477–0.761), 0.722 (95% CI, 0.575–0.840), 0.743 (95% CI, 0.601–0.854), 0.768 (95% CI, 0.625–0.877), and 0.706 (95% CI, 0.559–0.828), respectively. The UCSF criteria was entered into a multivariate analysis because it showed highest AUC value. There were significant correlations between T-bwSUVmax/L-bwSUVmean and T-SULmax (r = 0.639, *p* < 0.001), T-bwSUVmax/L-bwSUVmean and T-SULmax/L-SULmean (r = 0.730, *p* < 0.001), and T-SULmax and T-SULmax/L-SULmean (r = 0.730, *p* < 0.001). T-SULmax/L-SULmean was included in multivariate analysis because it had the highest value on AUC analysis. Multivariate analysis identified that the UCSF criteria (*p* = 0.004) and T-SULmax/L-SULmean (*p* = 0.003) were significant factors for the prediction of recurrence (Table 5). The AUC of the UCSF criteria with added high T-SULmax/L-SULmean models for prediction of tumor recurrence was 0.818 (95% CI, 0.682–0.914). Although the AUC of T-SULmax/L-SULmean showed a slightly higher value, there was no significant difference in the AUCs for prediction of tumor recurrence between the T-SULmax/L-SULmean and UCSF criteria (*p* = 0.339), between the T-SULmax/L-SULmean and UCSF criteria with added high T-SULmax/L-SULmean (*p* = 0.493), and between the UCSF criteria and UCSF criteria with added high T-SULmax/L-SULmean (*p* = 0.473).

## 4. Discussion

This study demonstrated that the metabolic parameters, especially T-SULmax/L-SULmean, measured by ^18^F-FDG PET/CT, were significant prognostic factors for the prediction of tumor recurrence in patients who underwent primary LDLT without pre-LT LRT for HCC. The performance of SUL-based metabolic parameters was superior to that of their bwSUV-based counterparts. Moreover, T-SULmax/L-SULmean showed the best performance for recurrence prediction with a cut-off value of 1.81. The comparison with other clinicopathological factors using multivariate analysis revealed that T-SULmax/L-SULmean was one of the two significant factors for the prediction of tumor recurrence, with the UCSF criteria being the other predictor. Although there was no significant added value of T-SULmax/L-SULmean to the UCSF criteria for the prediction of tumor recurrence, the UCSF criteria may have limitations in pre-LT patient selection due to the limited value of other radiographic examinations in pre-LT evaluation. Therefore, the T-SULmax/L-SULmean of pre-LT ^18^F-FDG PET/CT is considered to be more applicable than the UCSF criteria for selecting pre-LT patients.

^18^F-FDG PET/CT, which is a well-known imaging modality reflecting the glucose metabolism of the tumor, is used not only for tumor staging but also for detecting extrahepatic metastasis. Moreover, several recent studies have reported the utility of pre-LT ^18^F-FDG PET/CT for predicting tumor recurrence in patients with HCC and the selection of suitable candidates for LT [19,20,21,22,31,32,33,34]. These studies employed visual assessment (tumoral ^18^F-FDG uptake demarcated from the surrounding background liver) and various metabolic parameters, including the T-bwSUVmax and bwSUV ratio of the tumor to normal reference regions, such as the liver or inferior vena cava (IVC). Although the bwSUVmax is widely used for the evaluation of malignancies, it is susceptible to limitations arising from various biological and technological factors [35,36]. Therefore, the uptake ratio of the tumor to the normal reference region is more commonly used as an effective parameter than T-bwSUVmax alone [19,20,22,34]. The performance of the ratios of the tumor to the background liver for the prediction of tumor recurrence was superior to that of bwSUVmax or SULmax alone, based on the comparison of the AUC in this study. Thus, the ratio of the tumor to the normal reference region is considered to be more appropriate for the prediction of HCC recurrence than the maximum uptake value of the tumor.

Several patients with HCC have underlying liver diseases, such as viral hepatitis or chronic alcoholism-related disorders, which may affect ^18^F-FDG uptake in the liver. Previous studies reported that the value of the L-bwSUVmean was higher in patients with chronic hepatitis and fibrosis than that in the LC or normal control group [37,38]. However, these studies reported a relatively wider range of average values for the normal liver, which exhibited a slight overlapped with those in liver disease. All patients in the present study had underlying liver disease with or without LC and the value of the hepatic metabolic parameters tended to be lower in the LC group compared to the non-LC group, although this difference lacked statistical significance. Using the background liver as a normal reference is thought to be a good idea since HCC develops in the liver with underlying disease and glucose metabolism in HCC is based on glucose metabolism in the diseased liver. Moreover, in our study as well as other studies, the ratio of the tumor to normal reference region (with the background liver as the normal reference region) showed good performance for predicting tumor recurrence [19,20,22,32,34]. A recent study reported that an alternative method using the IVC as the normal reference region showed a slightly higher predictive value compared to the background liver [20]. In that study, cylindrical VOIs of 1 cm^3^ were drawn at 3 levels of the abdominal IVC and the bwSUVmean was obtained. Subsequently, the ratio of tumor-to-IVC activity was calculated, which showed the best performance for predicting tumor recurrence with a cut-off value of 1.16. However, a volume of 1 cm^3^ is relatively small, which makes it difficult to avoid the partial volume effect [39]. Another study showed that the bwSUVmean of the aorta in patients with normal liver parenchyma overlapped with that of patients with liver fibrosis/cirrhosis [37]. Therefore, further large well-controlled studies are needed to investigate the ideal normal reference region.

The BMI is widely known to be a significant factor affecting ^18^F-FDG uptake in both malignant and normal tissues. Previous studies have reported that BMI is a significant factor affecting ^18^F-FDG uptake in the liver, along with the injection-to-scan time interval and serum glucose level [40,41]. ^18^F-FDG uptake in the liver was significantly elevated in patients with a higher BMI. This may be attributed to the fact that the injection dose of ^18^F-FDG is determined by the patient’s body weight, and the ^18^F-FDG uptake in fat tissue is very low in the fasting state, resulting in increased uptake in non-fatty tissue [41]. Since the liver is used as the normal reference region for predicting tumor recurrence, it is necessary that the analysis of these predictive factors should reflect the effect of BMI. A recent study reported SUL-based parameters obtained from the liver did not differ significantly between patients with obesity and those with normal BMI [24]. In the present study, the SUL-based metabolic parameters in the liver did not differ significantly between the overweight/obese and normal BMI groups, and the SUL-based metabolic parameters showed superior performance over the bwSUV-based metabolic parameters. Furthermore, T-SULmax/L-SULmean had the highest AUC and a substantially large hazard ratio on the multivariate analysis. This can be explained by the fact that the T-bwSUVmax was 19% higher than the T-SULmax, and the L-bwSUVmax and L-SULmax, and L-bwSUVmean and L-SULmean differed by 20–29%. These differences were greater in the liver compared to the tumor and in the overweight/obese group than that in the normal BMI group. Thus, the tumor-to-background-liver ratio was thought to demonstrate better predictive performance because it could further emphasize the uptake of ^18^F-FDG in the tumor itself. Therefore, it is necessary to consider the BMI when using the liver as a normal reference region for predicting tumor recurrence.

There are several limitations to this study. First, this was a retrospective study and there was some heterogeneity in the patient management protocols, especially in post-LT management. The variation in management protocols could have introduced bias, even if it was not significant. Second, LDLT was performed even in high-risk patients beyond the scope of the clinical criteria, who were included in this study. However, we enrolled consecutive patients and performed routine ^18^F-FDG PET/CT scanning before LDLT in patients with HCC. Third, the present study was performed using data from a single center and selection bias could have influenced the survival analysis. Fourth, two different PET/CT systems were used, which could have affected the metabolic parameters. We used the same dedicated vendor-supplied workstations to minimize the effect of any differences between the devices. Finally, most tumor recurrences were clinically diagnosed using imaging studies and some cases were not pathologically confirmed, particularly those of suspected bone metastasis.

## 5. Conclusions

This study demonstrated that metabolic parameters, especially SUL-based metabolic parameters, obtained from ^18^F-FDG PET/CT are significant independent factors for predicting tumor recurrence after primary LDLT for HCC in patients who have not received pre-LT LRT. T-SULmax/L-SULmean was one of the most significant factors of survival analysis compared to various clinicopathological factors. Therefore, routine ^18^F-FDG PET/CT is recommended for the pre-LT evaluation of HCC not only for staging but also for predicting tumor recurrence. This policy could help to select suitable candidates for LT.

## Figures and Tables

**Table 1:** Clinicopathological characteristics of patients.

Variables	Overall	Non-Recurrence	Recurrence	*p* Value
Patients (*n*)	49	33	16	
Age, mean (years)	53.8 ± 5.8	54.0 ± 5.8	53.4 ± 6.0	0.920
Sex (female/male)	9/40	7/26	2/14	0.698
MELD score	10.3 ± 4.1	10.5 ± 4.7	10.0 ± 2.9	0.762
Child–Pugh score	6.3 ± 1.8	6.3 ± 1.9	6.4 ± 1.8	0.862
Body mass index, mean	23.7 ± 3.0	24.4 ± 2.9	22.2 ± 2.9	0.038 *
Etiology				0.162
HBV	37	22	15	
HCV	5	5	0	
Neither HBV nor HCV	7	6	1	
Liver cirrhosis (negative/positive)	18/31	13/20	5/11	0.754
Ascites (negative/positive)	39/10	28/5	11/5	0.261
AFP, median (ng/mL)	37.1 (range, 1.0–147,390.7)	28.0 (range, 1.3–44,848.8)	73.2 (range, 1.0–147,390.7)	0.084
PIVKA-II, median (mAU/mL)	52.0 (range, 9.0–18,361.2)	37.4 (range, 9.0–9853.0)	560.0 (range, 15.0–18,361.2)	0.072
Cold ischemia time (min)	60.7 ± 39.6	58.4 ± 41.1	61.9 ± 38.2	0.358
Warm ischemia time (min)	36.2 ± 14.5	35.4 ± 13.2	36.6 ± 15.8	0.581
Estimated blood loss (mL)	3412 ± 2688	3235 ± 1337	3478 ± 3521	0.159
GRWR	1.11 ± 0.23	1.08 ± 0.23	1.18 ± 0.23	0.285
T-stage (1/2/3/4)	24/15/7/3	21/10/2/0	3/5/5/3	0.001 *
Tumor number, mean	2.3 ± 1.5	2.0 ± 1.3	2.9 ± 1.7	0.063
Largest tumor size, mean (cm)	4.5 ± 4.7	3.0 ± 2.0	7.5 ± 7.0	<0.001 *
Microvascular invasion (negative/positive)	46/3	33/0	13/3	0.030 *
Milan criteria (within/beyond)	23/26	22/11	1/15	<0.001 *
UCSF criteria (within/beyond)	33/16	28/5	5/11	<0.001 *
Up-to-seven criteria (within/beyond)	32/17	26/7	6/10	0.009 *

* Statistically significant; MELD, model for end-stage liver disease; HBV, hepatitis-B virus; HCV, hepatitis-C virus; AFP, alpha fetoprotein; PIVKA-II, prothrombin-induced by vitamin-K absence or antagonist-II; GRWR, graft to recipient weight ratio; UCSF, University of California, San Francisco.

**Table 2:** Comparison of metabolic parameters according to body mass index.

Variables	Overall (*n* = 49)	*p* Value	BMI ≤ 25 (*n* = 33)	*p* Value	BMI > 25 (*n* = 16)	*p* Value
T-bwSUVmax	3.68 ± 2.62	<0.001 *	4.07 ± 2.99	<0.001 *	2.89 ± 1.41	0.008 *
T-SULmax	3.09 ± 2.10		3.38 ± 2.39		2.48 ± 1.16	
L-bwSUVmax	2.53 ± 0.47	<0.001 *	2.44 ± 0.41	<0.001 *	2.71 ± 0.55	0.001 *
L-SULmax	2.11 ± 0.43		2.13 ± 0.39		2.07 ± 0.50	
L-bwSUVmean	1.98 ± 0.50	<0.001 *	1.94 ± 0.47	<0.001 *	2.08 ± 0.55	<0.001 *
L-SULmean	1.53 ± 0.40		1.56 ± 0.38		1.45 ± 0.43	

* Statistically significant. BMI, body mass index; T-, tumor; L-, liver; SUV, standardized uptake value; bwSUV, SUV normalized by total body weight; bwSUVmax, maximum value of bwSUV; bwSUVmean, mean value of bwSUV; SUL, SUV normalized by lean body mass; SULmax, maximum value of SUL; SULmean, mean value of SUL.

**Table 3:** ^18^F-fluorodeoxyglucose positron emission tomography/computed tomography findings according to recurrence.

Variables	Overall	Non-Recurrence	Recurrence	*p* Value
Visual findings (negative/positive)	31/18	23/10	8/8	0.217
T-bwSUVmax	3.68 ± 2.62	3.26 ± 1.30	4.56 ± 4.14	0.654
T-bwSUVmax/L-bwSUVmax	1.49 ± 1.00	1.29 ± 0.54	1.92 ± 1.51	0.070
T-bwSUVmax/L-bwSUVmean	1.90 ± 1.09	1.66 ± 0.59	2.39 ± 1.63	0.050
T-SULmax	3.09 ± 2.10	2.56 ± 1.10	4.16 ± 3.12	0.009 *
T-SULmax/L-SULmax	1.46 ± 0.92	1.19 ± 0.41	2.01 ± 1.36	<0.001 *
T-SULmax/L-SULmean	2.02 ± 1.11	1.65 ± 0.53	2.77 ± 1.58	<0.001 *

* Statistically significant. T-, tumor; L-, liver; SUV, standardized uptake value; bwSUV, SUV normalized by total body weight; bwSUVmax, maximum value of bwSUV; bwSUVmean, mean value of bwSUV; SUL, SUV normalized by lean body mass; SULmax, maximum value of SUL; SULmean, mean value of SUL.

**Table 4:** Receiver-operating characteristic curve analysis of ^18^F-fluorodeoxyglucose positron emission tomography/computed tomography metabolic parameters for prediction of recurrence.

Variables	Cutoff Value	AUC	95% CI	Sensitivity (%)	Specificity (%)	Youden Index	*p* Value
T-bwSUVmax	4.60	0.540	0.391–0.683	25.0	93.9	0.189	0.676
T-bwSUVmax/L-bwSUVmax	1.11	0.661	0.512–0.790	68.8	63.6	0.324	0.056
T-bwSUVmax/L-bwSUVmean	1.91	0.674	0.525–0.801	50.0	81.8	0.318	0.047 *
T-SULmax	2.78	0.732	0.586–0.848	68.8	69.7	0.385	0.003 *
T-SULmax/L-SULmax	1.05	0.820	0.684–0.915	100.0	57.6	0.576	<0.001 *
T-SULmax/L-SULmean	1.81	0.850	0.720–0.936	87.5	78.8	0.663	<0.001 *

* Statistically significant. AUC, area under the receiver-operating-characteristic curve; T-, tumor; L-, liver; SUV, standardized uptake value; bwSUV, SUV normalized by total body weight; bwSUVmax, maximum value of bwSUV; bwSUVmean, mean value of bwSUV; SUL, SUV normalized by lean body mass; SULmax, maximum value of SUL; SULmean, mean value of SUL.

**Table 5:** Results of univariate and multivariate analyses for prediction of recurrence-free survival.

	Univariate Analysis			Multivariate Analysis		
Variables	HR	0.950	*p* Value	HR	95%	*p* Value
Sex (female vs. male)	1.798	0.409–7.917	0.438			
MELD score	0.981	0.856–1.125	0.981			
Child–Pugh score	1.008	0.767–1.324	0.955			
BMI (≤25 vs. >25)	0.648	0.209–2.015	0.454			
Liver cirrhosis (negative vs. positive)	1.304	0.452–3.758	0.624			
Ascites (negative vs. positive)	2.045	0.709–5.894	0.185			
AFP (≤150 vs. >150 ng/mL)	1.823	0.677–4.911	0.235			
PIVKA-II (≤100 vs. >100 mAU/mL)	3.090	1.144–8.345	0.026 *	1.262	0.426–3.742	0.675
T-stage (T1/2 vs. T3/4)	5.346	1.996–14.317	0.001 *			
Tumor number (≤3 vs. >3)	2.982	1.081–8.225	0.035 *			
Tumor size (≤5 cm vs. >5 cm)	3.661	1.311–9.946	0.013 *			
Microvascular invasion (negative vs. positive)	6.867	1.900–24.819	0.003 *	1.005	0.255–3.954	0.995
Milan criteria (within vs. beyond)	15.153	1.999–114.858	0.009 *			
UCSF criteria (within vs. beyond)	6.363	2.202–18.384	0.001 *	5.905	1.783–19.552	0.004 *
Up-to-seven criteria (within vs. beyond)	3.431	1.245–9.451	0.017 *			
PET visual (negative vs. positive)	1.844	0.690–4.932	0.223			
T-bwSUVmax (≤4.6 vs. >4.6)	3.052	0.971–9.595	0.056			
T-bwSUVmax/L-bwSUVmax (≤1.11 vs. >1.11)	2.842	0.985–8.198	0.053			
T-bwSUVmax/L-bwSUVmean (≤1.91 vs. >1.91)	2.806	1.050–7.497	0.040 *			
T-SULmax (≤2.78 vs. >2.78)	3.553	1.231–10.254	0.019 *			
T-SULmax/L-SULmax (≤1.05 vs. >1.05)	53.321	0.914–3111.518	0.055			
T-SULmax/L-SULmean (≤1.81 vs. >1.81)	11.962	2.713–52.747	0.001 *	11.142	2.298–54.017	0.003 *

* Statistically significant. MELD, model for end-stage liver disease; BMI, body mass index; AFP, alpha fetoprotein; PIVKA-II, prothrombin-induced by vitamin-K absence or antagonist-II; UCSF, University of California, San Francisco; T-, tumor; L-, liver; SUV, standardized uptake value; bwSUV, SUV normalized by total body weight; bwSUVmax, maximum value of bwSUV; bwSUVmean, mean value of bwSUV; SUL, SUV normalized by lean body mass; SULmax, maximum value of SUL; SULmean, mean value of SUL.

## Data Availability

The data presented in this study are available on request from the corresponding author.
